# Supplementary material for: Are carbon emissions trading and green financial instruments synergistic? -Comprehensive quantitative research based on content analysis
Source: PLoS One. 2024 Mar 7;19(3):e0298601. doi: 10.1371/journal.pone.0298601 (PMC10919601; doi:10.1371/journal.pone.0298601)
Supplement: S1 Data — (ZIP) [file pone.0298601.s001.zip › Data/地级市Z_F工作报告.zip!/╣π╢1⁄2/╖≡╔╜╩╨/└┤╫╘╣1⁄2╓┌║┼í╛┬φ┐╦╩2╛▌═°í┐_2007.docx]

各位代表：

　　我代表佛山市人民政府向大会作政府工作报告，请予审议，并请政协各位委员和其他列席人员提出意见。

本届政府任期工作回顾

　　本届政府任职时期，是我市行政区划调整后全面推进新的行政管理体制的关键时期，也是我市圆满完成“十五”计划、启动“十一五”规划的重要时期。2003年以来，在省委、省政府和市委的正确领导下，在市人大及其常委会和市政协的监督支持下，市政府坚持以邓小平理论和“三个代表”重要思想为指导，树立和落实科学发展观，抢抓机遇，团结实干，改革创新，加快推进产业强市、文化名城、现代化大城市与富裕和谐佛山建设，推动经济和社会各项事业又好又快发展，城市综合竞争力不断提升，全面完成“十五”计划各项指标和市十二届人大会议确定的各项目标任务。

　　一、经济发展步入新阶段，综合实力迈上新台阶

　　2003年以来，全市生产总值年均增长18.6%，2006年，全市生产总值2926.72亿元，增长19.3%；按常住人口计算，人均生产总值50207元（折合6276美元），增长18.2%；农业总产值163.77亿元，增长4%；工业总产值7070.80亿元，增长23.4%；地方财政一般预算收入157亿元，增长20%；税收总额440亿元，增长19.2%；全社会固定资产投资901.43亿元，增长19.1%；社会消费品零售总额775.95亿元，增长19.8%。东南部实现高基数上的高增长，西北部实现跨越式发展。顺德区成为全国首个县域GDP突破千亿元的县（区）。

　　二、产业强市建设加快推进，经济结构调整成效显现

　　产业结构进一步优化。农业园区化、产业化、现代化进程加快，全市现有国家级农业龙头企业1家、省级农业龙头企业4家；海峡两岸农业合作试验区建设开局良好。工业结构呈现出适度重型化、高级化趋势，规模以上轻重工业比例由2002年的57.7∶42.3调整为2006年的48∶52；汽车配件、液晶显示器和医疗器械等新兴产业不断发展；陶瓷、纺织和有色金属等传统产业改造提升加快。以物流业为重点的现代服务业快速发展，成功举办了三届佛山（国际）物流合作洽谈会，共签约项目276个，签约金额560多亿元；现代营销方式和新型零售业态不断涌现，会展经济发展迅速。节约型社会建设有序推进，清洁、安全、节能生产得到推广，万元GDP能耗下降4.5%。内外源型经济协调发展。2003年以来，民营工业产值年均增长30%以上，2006年全市规模以上民营企业实现工业产值3432.00亿元，增长32.5%，占全市规模以上工业总产值的55%。企业参与联合国采购实现突破，一批企业注册成为联合国采购供应商。2006年，全市合同利用外资17.7亿美元，增长14.66%；实际利用外资11.4亿美元，增长22.44%。引进奇美薄膜液晶显示器、英威达氨纶纤维、本田变速箱等一批科技含量高、带动能力强的龙头项目。目前有37家世界500强企业在佛山投资了70个项目，49家国内500强企业投资了66个项目。2006年，全市进出口总值309.8亿美元，增长20.4%；其中出口总值211.4亿美元，增长23.7%。科技创新能力不断增强。建立了一批省级研究院、国家级质检基地等公共研发服务平台。全市现有国家级特色产业基地16个，国家火炬计划重点高新技术企业41家，国家级企业技术中心3个，省级工程中心59个，省级技术创新专业镇30个，院士工作室6个，博士后工作站19家。2006年，全市高新技术产品产值1600亿元，占规模以上工业总产值的25.6%。全市专利申请量和授权量均居全省前列。我市荣获“全国科技进步先进市”称号和被确定为“全国专利工作试点城市”。实施名牌战略成效显著。全市现有中国驰名商标16件，中国名牌产品53个，广东省著名商标151件，广东省名牌产品157个。一批区域品牌落户我市。美的、格兰仕、新中源成为广东省重点扶持争创世界名牌产品的龙头企业。

　　三、各项重点项目建设全面推进，现代化大城市框架初现

　　规划龙头作用得到加强。坚持以规划引领经济社会发展和城市建设，编制并实施我市现代化大城市发展各项规划，确定走组团式现代化大城市发展道路。建立健全城市规划管理体制，完善各项规划管理制度，统一规划管理标准，初步形成层次分明、有机衔接、科学合理的城市规划体系。组团城市及重点工程建设全面推进。把握承办第七届亚洲艺术节和第十二届省运会的机遇，全面启动构建现代化大城市框架和雏形的“十大工程项目”建设。组团城市建设稳步推进，中心组团新城区启动区建设初具规模。以“一环”为重点的21项交通基础设施建设不断加快，包括“一环”在内的15项工程全面建成通车；“禅桂新”10项路网工程如期完成；高速公路、港口航道建设进展顺利。公共交通加快发展，广佛交通路网逐步对接。承办亚艺节和省运会的重点场馆先后投入使用。电源电网建设不断加强，液化天然气（LNG）工程进入商业运营。城乡水利防灾减灾工程建设扎实进行，防洪排涝体系不断完善。全社会信息化水平不断提升，信息化综合指数达74.8%。经营城市机制基本形成，一批城市可经营项目推向市场。生态环境建设取得阶段性成果。创建国家环境保护模范城市工作扎实推进，大气污染治理、内河涌和珠江水环境综合整治取得明显成效，城市环境得到改善。全面创建国家卫生城市，通过国家爱卫会复查。全市共创建国家卫生镇15个、省卫生镇4个、省卫生村371个、省级生态示范区（镇、园、村）35个。城市管理工作得到加强。城市管理架构初步建立，城市管理制度逐步完善，城市管理模式和方法不断创新，城市管理综合执法得到加强。开展“城市管理年”活动，交通秩序和市容市貌整治取得较好成效。

　　四、文化名城建设扎实推进，社会各项事业不断进步

　　制订并实施《建设文化名城规划纲要》。全市群众文化四级网络基本形成，公共文化服务体系逐步完善。成功举办琼花粤剧艺术节、武术文化节、中国陶艺文化节、中国厨师节等系列活动。文化遗产保护、开发和利用工作得到加强，广东醒狮等6个项目入选首批国家级非物质文化遗产名录。成功承办亚洲文化部长论坛暨第七届亚洲艺术节。签署《佛山宣言》，城市形象和文化内涵进一步提升。成功承办广东省第十二届运动会和第五届省残运会。竞技体育水平跃上新台阶，群众性体育活动蓬勃开展，多元化全民健身服务体系不断完善。我市荣获“武术之城”称号。成功创建“广东省教育强市”。初步建立起比较完善的现代国民教育体系。建立和完善扶贫助学新机制。全市城乡全面实施免除义务教育阶段杂费。我市成为广东省教育现代化试点市。人才强市战略深入实施。2006年全市共引进在职人才1378名，接收毕业生18267名，其中中高级职称及硕士以上学历人员1222名。目前，全市人才总数达59万人。卫生、人口与计生事业全面发展。基本形成覆盖全市的公共卫生三级网络，卫生监督、疾病控制、医疗救治、妇幼保健、精神卫生、采供血安全、农村和社区卫生服务等工作取得新成效；食品、药品、农产品卫生安全监管得到加强。积极探索人口与计划生育管理服务新机制，全市户籍人口出生率9.08%。，自然增长率4.28%。

　　五、和谐社会建设稳步进行，人民生活水平不断提高

　　精神文明建设取得可喜成果。深入贯彻实施《公民道德建设实施纲要》，积极开展现代公民教育、社会主义荣辱观教育和未成年人思想道德建设活动，成功创建“广东省文明城市”。国防建设和民兵预备役、拥军优属工作进一步巩固和提高，我市第五次荣获“全国双拥模范城”称号。统筹城乡协调发展取得明显成效。颁布并实施农村工作“五十条”及首批九个配套文件，以农村“三化”解决“三农”问题取得阶段性成果。优化整合路桥收费站、公交、邮政、电信、旅游、传媒和教育等公共资源，推动城市基础设施向农村延伸，城市公共服务向农村覆盖，不断向“同城生活、同城便利、同城发展”的方向迈进。就业再就业工作取得新进展。城乡培训就业一体化进程加快，城乡就业服务体系逐步健全。2006年，全市共培训农村富余劳动力2.34万人，实现转移就业1.35万人，就业率为58%；全市累计促成城镇登记失业人员就业3.6万人，期末城镇登记失业率为1.8%。社会保障体系不断完善。全市城镇职工基本养老、工伤、失业保险全面实行市级统筹，积极推进基本医疗保险市级统筹。到2006年10月底，参加养老、失业、医疗、工伤保险人数分别为126.73万人、105.78万人、109.41万人、147.76万人。建立健全全征土地农村居民养老保险补贴制度和农村居民基本医疗保险制度。低保、五保、住房、法律援助、临时救济、自然灾害救济等救助机制不断完善。安全生产形势平稳。全国非公有制中小企业安全生产试点工作顺利完成，初步建立起安全生产监管长效机制。社会大局基本稳定。建立“五网络一中心”社会安全防控体系，深入开展严打整治斗争，积极推进科技强警工作，开展平安和谐社区创建活动。建立城市公共安全应急管理机制，成功抗击“非典”、防控高致病性禽流感等疫情，战胜百年一遇特大洪涝、台风灾害袭击。做好人民调解和信访工作，妥善处理社会各种矛盾。建立企业工资支付监控制度，大力清理拖欠工程款和农民工工资。进一步完善法律援助工作，维护外来务工人员合法权益。人民生活水平不断提高。2006年，全市城市居民人均可支配收入18850元，增长8.2%；农村居民人均纯收入8107元，增长7%；城乡居民储蓄存款余额2678.79亿元，增长13.6%。

　　六、民主法制建设逐步深入，政府理政能力不断增强

　　民主法制建设逐步深入。坚持向市人大及其常委会报告工作，重视和支持人民政协以及各民主党派、工商联履行政治协商、民主监督和参政议政职能。2003年至今，共办理人大代表议案、建议410件，政协委员提案765件，办复率100%。全面加强依法治市建设，完成“四五”普法工作。贯彻落实《行政许可法》和《全面推进依法行政实施纲要》，出台《佛山市人民政府工作规则》，开展相对集中行政处罚权试点工作，政府依法行政能力不断提高。政府自身建设不断增强。开展共产党员先进性教育活动和排头兵实践活动，政府理政能力得到提升。实行简政放权，先后向各区下放322项行政管理事项。深化行政审批制度改革，清理各种审批事项1400项。市与五区统一规范的行政服务体系初步建立。完成市、区两级政府机构改革，推进镇（街）、村（居）调整撤并，政府社会管理和公共服务职能得到加强。设立行政投诉中心，强化行政执法监察和效能监察。加强电子政务建设，推行政务公开，建设阳光政府。发挥新闻媒体舆论监督作用，建立政府部门新闻发言人制度，实行重大政策出台、重大项目决策前公众参与、专家论证制度。落实党风廉政建设责任制，市政府向社会公开提出并认真履行四项廉政承诺。建设行政审批电子监察系统，完善重大建设工程项目廉政监督检查机制。认真开展治理商业贿赂工作，深入开展纠风专项整治，严肃查处违法违纪案件。

与此同时，老年人、残疾人、妇女儿童事业，粮食储备、民族宗教、外事侨务、对台港澳事务、广播电视、人防、气象、档案、方志、物价、统计、扶贫等各项工作都取得新成绩。

　　各位代表！本届政府任职以来所取得的成绩，是全市人民在省委、省政府以及市委的正确领导下，与时俱进、同心同德、务实进取、努力拼搏的结果。在此，我谨代表佛山市人民政府，向辛勤工作在各条战线的广大干部群众，向给予政府工作大力支持的人大代表和政协委员，向中央、省驻佛山单位，向驻佛山人民解放军指战员、武警官兵和各民主党派、工商联、人民团体以及社会各界人士致以崇高的敬意！向所有参与、关心和支持佛山建设的港澳台同胞、海外侨胞、国际友人以及外来务工人员表示衷心的感谢！

过去几年政府工作虽然取得了一定成绩，但在经济社会发展中仍然存在一些矛盾和问题。主要是：自主创新力量不足，经济增长方式有待转变；发展资源约束矛盾突出，可持续发展能力有待提高；经济社会发展还存在着体制机制障碍；城市规划建设管理滞后于经济发展，生态环境有待进一步改善；政府职能转变还未完全到位；社会主义新村镇建设还有不少困难；城镇化进程中的“三农”问题亟需解决，劳资矛盾、社会不稳定因素仍需化解，和谐佛山建设任重道远，等等。对于这些问题，我们一定保持清醒头脑，高度重视，采取有力措施，切实加以解决。

主要工作体会

　　本届政府任职期间，不仅大力推进经济、文化和社会各项事业发展，而且在实践中升华了思想认识，提高了利用重要战略机遇期推动经济社会又好又快发展的自觉性，增强了贯彻落实科学发展观、构建和谐社会的能力。我们的主要体会是：

　　——必须坚持产业发展不动摇，夯实经济社会发展基础。我市的区位优势、经济发展和人文特点决定了必须坚持走“产业强市”之路。近年来我市经济实力不断跃上新台阶，关键在于坚持了区、镇是经济发展主战场不动摇。同时，不断深化改革，优化政务和营商环境，打造产业发展体制机制优势，充分调动各区发展经济的积极性，在全市形成“五虎争先，虎虎生威”新局面，我们还加强专业镇、工业园区和特色产业基地建设，实施“双轮驱动”战略，鼓励企业参与国际竞争，提升产业发展水平。实践使我们认识到，产业是佛山的命脉，产业兴则佛山兴，产业强则佛山强。我们要始终坚持把产业发展摆在全市经济社会发展优先地位，一切政策和措施的制订都必先有利于产业发展，任何规划、建设和管理都应该起到促进产业发展的作用，这样才能把佛山建设得更强更大。

　　——必须坚持走组团式城市发展道路，加快建设有现代化城市建筑标志，包容佛山产业特征，具有岭南水乡特点，体现深厚历史文化底蕴的现代化大城市。借鉴国内外城市规划建设先进理念，走组团式城市发展道路，构建现代化大城市新格局，是我们促进各种资源要素优化配置，推动城乡互动，提升区域综合竞争力的必需。要坚定不移地推进“2+5”城市组团建设，按照“统一规划、统一标准、统一安排，属地实施、属地管理、属地受益”的原则和“同城生活、同城便利、同城发展”的目标，加快组团式城市基础设施的无缝对接，密切我市与周边城市之间的区域联系，促进经济向组团式城市方式集聚和发展。要进一步确立“经营城市”理念，推动城市可经营性项目资本化管理、市场化经营。要注重城市文物遗迹保护和修缮，促进历史文化底蕴与现代城市气息相交融。要强化城市管理，改善城市环境，提升城市形象，把佛山建成产业重点突出、文化底蕴深厚、城市功能完善、组团联系紧密、生态环境良好、岭南水乡特色鲜明的现代化大城市。

　　——必须坚持区、镇是经济发展的主战场，不断壮大县域、镇级经济。在发展经济、解决“三农”问题、推进社会主义新村镇建设中，区、镇（街）是最直接的组织者和实施者。要充分认识区、镇是经济发展的主战场、城镇化建设的重要载体和干部锻炼成长的大平台。市政府及各部门要把精力集中在抓好全市重大规划的编制、重要政策的制订、公用事业和基础设施建设的统筹安排以及调研、策划、协调和监督管理上，把可以下放的权责尽量下放到基层，促进区域综合竞争力的提升和发展，从而推动我市产业发展和综合实力不断跃上新台阶。

　　——必须坚持城乡一元发展方向，统筹城乡经济、文化、社会协调发展。要逐步实现从城乡二元格局向城乡一元结构的跨越，关键是：要坚持以城带乡、以工促农、城乡互动，建立健全有利于改变城乡二元结构的体制机制，在行政决策、制定政策、出台文件、推动发展等工作中始终坚持城乡一元发展原则；要大力推进工业化、城市化、农业产业化进程，加快外源农业、生态农业、观光农业和外汇农业发展；要积极发挥第二、三产业在吸纳城乡劳动力就业和促进城乡生产要素融合方面的重要作用，以产业化支撑城镇化，以城镇化带动城乡一体化；要统筹城乡基础设施建设，推动城市基础设施向农村延伸，城市公共服务向农村覆盖，城市文明向农村辐射，使农村居民真正享受到与城市居民同样的环境和服务，促进城乡加速融合，最终形成城乡一体化发展格局。

　　——必须坚持推进社会主义和谐佛山建设，使发展成果更多地惠及老百姓。我们要以科学发展观为指导，以解决人民群众最关心、最直接、最现实的利益问题为重点，以实施“十项民心工程”为抓手，加快发展群众文化、基础教育、基本医疗和社会保障等社会事业，切实解决人民群众“低保”、“行路难”、“饮水难”、“读书难”、“看病难”、“住房难”等问题。要加大公共财政对民心工程的投入力度，加快建立健全覆盖城乡的基本社会保障体系和基本公共服务体系、就业服务体系以及外来工合法权益维护体系，保障人民群众在社会保险、社会救助、社会福利和医疗、住房、培训就业等方面的权益。要加快建立健全社会矛盾化解机制，充分发挥职能部门的主导作用、新闻媒体的桥梁作用、工青妇等人民团体的纽带作用和城乡基层组织的基础作用，形成党委领导、政府负责、社会协同、公众参与的社会管理格局，确保社会稳定和谐。

今后五年奋斗目标及2007年工作任务

　　今后五年是我市进一步贯彻落实科学发展观和构建社会主义和谐社会，全面建设产业强市、文化名城、现代化大城市与富裕和谐佛山的关键时期，也是我市全面实现“十一五”规划目标、率先基本实现社会主义现代化的重要时期。

　　今后五年，我市经济社会发展面临重大机遇期。从国际方面看，世界经济形势相对稳定，新一轮国际资本流动和产业调整转移进程加快，为我市引进先进技术和高端产业、拓展国际市场提供了宝贵机遇。从国内方面看，本世纪头二十年是我国经济的黄金发展期，我国加入WTO过渡期刚刚结束，市场开放将进一步扩大。同时，国家实行财政、货币“双稳健”政策，势必有利于推动我市经济的科学健康发展和国际化进程。从我市实际看，经过20多年的改革开放，产业对城市综合实力的支撑力、基础设施对经济社会发展的承载力、精神文化对城市发展的凝聚力、城市对社会财富的集聚力都有大幅度提升，为我市进一步提高自主创新能力、促进产业结构优化升级提供了保障。我们应在新的起点上，认清形势，紧盯目标，抢抓机遇，乘势而上，推动我市经济、文化、社会的全面发展、科学发展、集聚发展、创新发展与和谐发展。

　　今后五年政府工作的指导思想是：以邓小平理论和“三个代表”重要思想为指导，以科学发展观统领经济社会发展全局，以构建社会主义和谐社会为总目标，传承历史、开创未来，凝聚和调动全市人民的积极性和创造性，着力推进产业强市、文化名城、现代化大城市与富裕和谐佛山建设，全面增强佛山城市综合竞争力，推动佛山经济建设、文化建设、社会建设和政治建设更上一个新台阶。

　　今后五年经济社会发展的奋斗目标是：力争地区生产总值比2006年翻一番，人均生产总值达到一万美元以上，综合实力显著增强，人民生活水平进一步提高。经济增长质量和效益明显提高，经济增长方式有效转变，万元GDP能耗比“十五”期末降低20%左右，主要污染物二氧化硫排放总量减少43%。县域镇级经济实力不断增强，经济国际化步伐加快，市场经济体制进一步完善，组团式大城市经济显现。自主创新能力明显增强，科技进步贡献率达到55%以上，创新型城市逐渐形成。基础设施建设加快推进，现代化大城市框架更趋完善。文化事业文化产业加快发展，文化名城建设取得新进展。就业和社会保障体系不断完善，公共服务体系比较健全。城乡一元发展格局基本形成，有佛山特点的社会主义新村镇建设取得较大进展。民主法制、精神文明建设全面加强，社会稳定和谐，率先基本实现社会主义现代化。

　　今后五年，要坚持统筹区域协调发展，推动东南部地区和西北部地区利用各种资源、发挥各自优势创新发展、错位发展。支持禅城区加快老城区、旧物业和城中村改造，实施“优二进三”战略；支持南海区重返全国百强县（区）三甲之列；支持顺德区继续率先发展,加快工业化、城市化、国际化进程；支持高明区打造“山林水都”，实现和谐跨越；支持三水区合理利用空间发展优质产业，促进经济又好又快发展。

　　2007年是深入贯彻落实科学发展观、推进富裕和谐佛山建设的重要一年，也是新一届政府的开局之年。做好今年政府工作意义深远、责任重大。我们要全面贯彻落实党的十六届六中全会、中央经济工作会议和省委九届十次全会精神以及市第十次党代会工作部署，坚定不移地聚精会神搞建设，一心一意谋发展，推动全市经济和社会各项事业发展再上新台阶。

　　2007年主要预期目标是：生产总值增长12%；万元GDP能耗下降4.3%，主要污染物二氧化硫排放总量减少10%；全社会固定资产投资增长12%；社会消费品零售总额增长16%；地方财政一般预算收入增长12%；进出口总值增长12%；城市居民人均可支配收入和农村居民人均纯收入分别增长8%和5.5%；居民消费价格总指数控制在3%左右；新增城镇就业岗位7万个，城镇登记失业率控制在3.5%以内；人口自然增长率控制5.9%。以下。

为实现上述目标任务，我们将着力抓好以下六方面工作：

　　一、推动经济创新发展，全力打造产业强市

　　实施“三三三”产业发展战略。第一个“三”是指促进三大产业协调发展。通过产业结构调整和升级，对三大产业的比重进行合理调配，促进第一产业精细发展、第二产业优化发展、第三产业加快发展，三大产业关系进一步协调。第二个“三”是指促进支柱行业均衡发展。在第一、二、三产业中，每个产业重点扶持三个或以上的支柱行业，将其作为能够带动整个经济发展的战略行业，使其带领和推动佛山经济的持续健康快速发展。第三个“三”是指促进龙头企业加快发展。在第一、二产业的每个支柱行业中重点扶持三家或以上的产业关联度大、技术装备水平高、自主创新能力强、经济实力雄厚、带动能力强的规模龙头企业，鼓励其继续做大做强，充分发挥龙头企业对行业的示范带动作用，推进整个行业的技术进步和快速发展；在第三产业的支柱行业中重点扶持三家或以上的服务范围广、带动能力强、从业人员多或专业水平高的服务业龙头企业，鼓励其在提供优质和广泛的服务、促进生产发展、方便市民生活等方面发挥更大的示范带头作用。加快建立实施“三三三”产业发展战略的推进协调机制，制定并实施“三三三”产业发展战略的具体扶持政策，提升政府超前引领服务水平，在规划指导、园区建设、科技创新、信用担保、财政扶持等方面扶持支柱行业和龙头企业发展，不断增强产业综合竞争力。坚持把实施“三三三”产业发展战略与完善产业发展规划相结合，进一步优化产业区域布局，形成布局合理、分工协作、竞争有序的经济发展格局。

　　实施企业“五阶段”发展战略。包括夯实基础、创造品牌、抢注专利、制定标准、让他人为我做贴牌生产五个阶段。夯实基础就是要加快制定和完善推动中小企业发展的扶持政策，重点在技术创新、信息咨询、人才培训、信用担保、品牌建设、国际市场拓展等方面扶持众多中小企业发展，与“三三三”发展战略形成战略互补。创造品牌就是要实施品牌带动战略，出台扶持、奖励措施，鼓励企业争创名牌；做好中国世界名牌以及国家级、省级名牌的推荐、申报工作，支持技术创新专业镇和特色产业基地创立区域品牌。抢注专利就是要加强知识产权工作，完善专利激励机制，加大专利信息服务扶持力度，鼓励支持企业抢注专利，建立专利数据库；通过完善知识产权工作的配套政策措施，引导和鼓励企业提升知识产权创造、管理、保护和运用能力，推动企业技术成果知识产权化和拥有自主知识产权的技术成果产业化。制定标准就是要加快制定技术标准研制、资助、奖励和管理办法，支持建立技术标准研究测试机构和基地，鼓励和支持企业参与制定行业标准、国家标准和国际标准。让他人为我做贴牌生产就是要通过制定政策、科学超前引领服务，推动我市中国名牌产品企业加快体制、管理、技术和文化创新，最终创造出自己的标准，成为世界名牌，进行品牌输出，加大国内、国际市场的占有份额。

　　实施“双轮驱动”战略。全力推进民营企业与外资企业协调发展。认真落实民营经济发展的各项扶持政策，完善具体的配套措施，鼓励民营企业创新体制机制，引导民营企业加快建立现代企业制度。鼓励民营企业借助资本市场，走上市发展做强做大的道路。同时，继续加大招商选资力度，着力引进科技含量高、带动能力强、附加值高、符合环保要求的大项目。鼓励和支持外资企业增资扩产。加强集约园区建设，预留发展空间承接国内外战略投资者和大企业、大项目落户；改善投资环境，完善配套服务体系，争取使世界500强、跨国公司在佛山设立区域总部、研发机构和采购中心。充分发挥汽车配件、液晶显示器等龙头项目的带动作用，引入更多的生产企业、配套企业向产业链上下游延伸。坚持“引进来”与“走出去”相结合，同步发展。在大力引进项目、资金、技术、管理和高级人才的同时，加大“走出去”的力度，政府通过搞好服务，引领我市本土企业嵌入国际供应链，成为国际供应链中的重要一环。鼓励和引导有条件的企业对外投资和跨国经营，推动企业参加国际展会、参与联合国采购活动，走国际化发展道路；支持具有自主知识产权、自主品牌的商品出口。健全进出口风险预警机制，增强企业防范国际市场风险和应对国际贸易摩擦的能力。加强区域经贸合作。更加积极主动地融入广佛经济圈、珠三角城市群发展；进一步加强与港澳、泛珠三角及国内其他城市的经贸合作与文化交流。

　　实施自主创新战略。坚持走以市场为导向、以企业为主体、以人才为根本、以产业技术为重点的自主创新路子，把佛山建设成为重要的高新技术研发基地、成果转化基地和先进制造业基地。推进产业结构优化升级。坚持以节约能源资源和保护生态环境为切入点，继续把淘汰落后生产能力作为调整和优化产业结构的重要途径，把强化政府责任作为实现节能降耗和污染减排目标的关键环节，把完善市场调节机制作为节能降耗和污染减排的基本手段，把加强依法治理作为节能降耗和污染减排的重要保证，利用高新技术、先进适用技术和信息化改造提升陶瓷、纺织、家具和有色金属等传统产业；加快发展汽车配件、显示器件、电子信息、光机电一体化、生物医药、医疗器械、新材料、精细化工等新兴产业和高新技术产业。加强区域创新体系建设。健全完善研发平台，创建科技孵化器，完善创新服务体系，强化企业的技术创新主体地位，提升自主创新能力，促进科研成果向现实生产力转化。加强产学研合作。推动我市企业与高校、科研院所形成科技创新联盟，解决产业发展的重大技术瓶颈问题。坚持引进与消化吸收再创新相结合。通过政府规划引导、设立专项资金等措施，支持企业在创新上下功夫，实现自主创新能力的快速提升，促进产业向高级化发展。坚持创新人才培养与创新文化建设相结合，广泛开展科普活动，鼓励科技创新实践，增强自主创新能力，建设创新型佛山。

　　加快发展以物流业、会展业、商贸业为重点的现代服务业。增加服务业在三大产业中的比重。大力培育和发展与制造业相配套的生产性服务业。加快发展现代物流业，推进物流基地建设，发展壮大现代物流龙头企业。把握服务业全面开放的机遇，吸引外商直接投资物流、会展、金融、会计法律咨询、市场调查等服务业领域。充分发挥佛山产业优势，与国内、国际专业协会合作举办大型产业会展，推动会展业专业化、市场化、品牌化和国际化；通过品牌会展业联动举办高标准的国际化会议和论坛。运用现代经营方式和信息技术改造提升商贸、餐饮、金融、旅游、中介、社区服务等为生活服务的现代综合服务业。加强对房地产市场的引导和调控，促进房地产业健康持续发展。

　　加快经济体制改革步伐。继续深化国有企业产权、公用事业体制、投融资体制等领域改革，进一步完善国有资产监管体系。打造新的平台，把市场配置资源机制引入经济发展和城市建设的各个领域。

　　二、坚持以规划为引领，提升现代化大城市建设水平

推进城市立体规划。按照现代化大城市的要求，把佛山作为一个城市整体，实行地下、地面、空中的立体规划，提升城市规划、建设和管理水平。抓紧修编全市土地利用总体规划，十分珍惜、合理利用土地，加强耕地保护。按照城市总体规划深化和完善各组团规划和专项规划，突出中心组团的辐射和带动作用，实现各组团规划和各专项规划的有效衔接。强化城市信息化规划作用，建设“数字佛山”。

　　全面加快组团式城市建设。将加强现代城市标志性建筑、包容佛山产业特征、营造岭南水乡生态特点的现代化大城市建设与开展历史文物保护开发有机结合，规划建设一批人文景观和建筑精品。健全组团城市建设推进协调机制，加快“2+5”城市组团、尤其是两个百万人口城市组团建设。重点加快中心组团新城区行政、商务、金融、文化、信息、展览等功能区建设，增强中心组团的引领和辐射带动作用。加大对跨区组团规划建设的统筹力度，协调处理好跨区组团市政设施配套、交通基础设施建设、城镇功能布局等的衔接，促使组团城市建设早见成效。健全经营城市机制，盘活城市资源，推动更多城市可经营性、可资本化资源走向市场。

　　加强以广佛地铁为重点的城市基础设施建设。加快完善城际快速轨道交通规划,建立健全广佛地铁建设协调机制,创新投融资体制，充分利用国内外资源，全力推进广佛地铁工程建设,力争今年尽早全面动工。抓紧编制并实施全市综合交通规划，促进城市交通可持续发展。加快完善与“一环”相连接的各区路网建设,包括“一环”东路南延线、北延线，以及广明高速、禅西大道、兴朗大桥、中心组团新城区道路网建设，推进全市区域间城市基础设施的对接。启动新一批干线道路工程项目建设。加快推进广佛两市道路系统衔接规划的实施，今年重点抓好与广州平南高速（南大干线）、沙湾干线、石壁大道、黄榄干线等四条道路的衔接。进一步整合港口资源，推进水路交通建设。积极配合做好广珠城际快速轨道交通、武广客运专线、广茂铁路扩能等铁路项目以及九条高速公路项目建设。实施公交发展优先政策，加快构建现代公交体系，全面提升我市公交运输效率。按照电力系统专项规划，科学布局电源电网项目，重点加强110千伏和220千伏输变电站建设。抓紧实施全市供水系统专项规划，有效整合城乡供水资源。加快第二饮用水源地的建设。继续推进液化天然气（LNG）工程建设。加快主干内河涌综合整治和电排站建设，进一步提升城市防洪排涝能力。

　　加快推进城市道路六级标准化和城市绿化建设。将城市道路和绿化纳入整体规划，把城市道路划分为城市快速路、主干道、次干道、支路、景观步行道及其他道路交通等六种交通形式，根据我市气候特点、水文土质条件，结合佛山生态水乡环境，在各级道路上选择、搭配相适宜的树种、花草，推进道路标准化和绿化建设。认真实施全市城市绿地系统、景观生态及林业生态发展规划，加快包括道路、居住小区、休闲地及林地等在内的城市绿化进程。大力推进生态示范社区、生态示范镇（村、园区）建设。实施“青山、绿地、蓝天、碧水”工程，力争用三年左右时间，实现“绿色佛山”目标，把佛山建成更加适合生活居住和创业发展的花园城市。

　　加强环境治理和保护。全面实施环境保护与生态建设“十一五”规划。继续认真抓好创模改进工作，确保创模目标的早日实现。以“一突破两改善”为主要内容，大力推进水环境和大气环境的综合整治。严格实施污染物排放总量控制制度，确保污染物排放总量逐年削减。积极推进节约型社会试点城市建设，突出抓好土地、水、能源和重要资源的节约和合理利用，加大对旧工业园区、旧城区、旧物业的改造提升。严格建设项目环保准入，落实环保工作“三个一律”。建立健全各级政府环保责任考核机制和责任追究制，强化环境　　监督执法和污染源在线监控。加强环保宣传，提升全社会环保意识。

加强城市管理工作。按照现代化大城市管理思路，推进城市管理向社会化、综合化、现代化、法制化转变。继续推动城市管理重心下移，强化区级城管职能。加大城市综合执法和整治力度，着力整治市容“脏、乱、差”和交通拥堵问题。加强“一环”沿线土地控制与监管，抓紧按规划实施。积极探索建立城市管理综合信息平台，建设“数字化城管”，提高城市管理水平，努力营造安全、有序、文明的城市环境。

　　三、加快推进文化名城建设，提升城市文化竞争力

着力构建公共文化服务体系。加快推进联合图书馆、数字图书馆、文化信息资源共享、有线数字电视整体平移、广播电视“村村通”、农村数字电影放映等工程建设，继续完善覆盖全市的流动演出网等六大文化服务网络，为市民提供丰富、优质的公共文化服务。通过加快岭南水乡特色文化建设，促进粤剧、陶艺、武术、醒狮、龙舟、秋色、美食、行通济等民俗文化的传承发展，提升文化名城的时代特色。抓好省历史文化名镇、名村试点建设。重视古村镇、古街区、历史名人故居、历史事件遗址的发掘保护和综合利用，做好祖庙修缮工作。

　　大力发展文化产业。落实促进文化产业发展的各项政策措施，扶持广电、报业、出版、网络媒体、印刷复制、文化旅游等产业加快发展，推进全国文化产业示范基地建设，培育壮大若干文化龙头企业，打造文化产业品牌。探索举办大型主题文化旅游活动，完善城市旅游环境。规范、活跃文化市场，提升文化市场监管水平。

　　积极培育和谐文化。加强社会主义核心价值体系建设。以实施《公民道德建设实施纲要》为主线，深入开展社会主义荣辱观教育、现代公民教育和未成年人思想道德教育活动，广泛开展社会公德、职业道德、家庭美德教育，大力提倡志愿服务，在全社会形成知荣辱、讲文明、促和谐的良好风尚。不断丰富和提升以“创新有为、崇德守法、求真务实、开放兼容”为主要内涵的佛山人精神，培养全体市民的现代化大城市意识。落实创建全国文明城市各项要求，深入开展文明村镇（街道）、文明单位创建活动。巩固和发展拥军优属工作，继续做好固强补弱工作，争创“全国双拥模范城市”六连冠。加强人文关怀和心理健康教育，塑造自尊自信、理性平和、积极向上、友爱包容的社会心态，增强市民的幸福感。

　　四、维护市民切身利益，构建富裕和谐佛山

　　推进教育均衡协调发展。坚持把教育放在优先发展的战略地位，巩固教育强市成果，推进教育现代化建设。进一步调整优化教育布局，加快整合教育资源，大力发展优质教育。加快发展学前教育，高水平、高质量普及九年义务教育和高中阶段教育，逐步缩小城乡、区域教育发展差距，推动公共教育协调发展。大力发展职业教育，促进职业教育与产业发展需求相对接；积极发展高等教育，着力抓好佛山市大学园区建设；扶持和规范发展民办教育，推进社区教育和成人教育，进一步完善现代国民教育体系和终身教育体系。在全市实施免除义务教育阶段杂费的基础上，今年秋季起对本市户籍的义务教育阶段学生免收书本费。继续完善扶贫助学机制，保障贫困家庭子女受教育的权利。妥善解决非户籍常住人口子女就学问题。推动教育国际化进程，全面提升教育综合实力。

　　完善城乡统筹就业和社会保障体系。继续完善和落实扶持就业再就业各项优惠措施，积极支持自主创业、自谋职业。规范用工管理，依法处置恶意拖欠工资问题，保护劳动者特别是外来务工人员合法权益。完善全市统一的基本养老、失业、工伤等社会保险制度，提高社会保险市级统筹水平，扩大社会保险覆盖面。认真落实城镇职工基本医疗保险全市统筹的政策，进一步提高医疗保障水平。完善农村基本医疗保险和全征土地农村居民养老保险补贴制度，落实城乡低保统一标准的政策，提高农村居民基本生活保障水平。完善住房保障制度，有步骤地解决低收入群众的住房问题。健全社会救助体系，完善市、区两级慈善机构，鼓励社会力量兴办福利和慈善事业。发展残疾人事业，提高残疾人社会保障水平。

　　提升公共卫生和医疗服务水平。加强疾病预防控制、医疗救治和卫生监督执法能力建设，大力推进农村三级卫生服务网络和以社区卫生服务为基础的新型城市卫生服务体系建设，健全城乡医疗卫生服务体系。落实好省提出的一个行政村建设一个医疗站、配备一名医生的规定，着力解决“看病难、看病贵”问题。加强医院和医药市场监管，重点整治非法行医、药价虚高、制售假劣药品等问题。加强食品、药品、农产品、餐饮卫生监管，保障人民群众健康安全。完善医疗服务信息公开制度，加强医德医风建设，构建和谐医患关系。

　　加强社会维稳综治工作。积极推进社会治安综合治理，努力排查化解各类矛盾纠纷。继续加强治安重点地区和突出治安问题的集中整治，重拳打击涉黑势力和团伙犯罪以及“两抢一盗”等多发性犯罪，扫除黄赌毒等社会丑恶现象，增强人民群众的安全感。巩固和推广科技强警示范市建设成果，全面推进平安佛山建设。强化政权意识和国家安全意识，严密防范和打击境外敌对势力的各种渗透破坏活动，进一步完善国家安全人民防线。强化对民间组织的规范管理。加强对外来务工人员、流动人员和出租屋的管理服务。做好人民调解、司法调解、法律援助和信访工作，着力解决群众反映强烈的问题，确保社会稳定和谐。

建立健全公共安全防控体系。建立政府统一领导的应急联动指挥系统，健全应急预案体系，做好对公共卫生事件、环境污染、生产安全事故、重大自然灾害等监测、预警预报和应急处置工作。完善安全生产监管机制，强化安全生产、消防安全责任制，防范和化解重特大安全事故。严格枪支弹药、易燃易爆物品、剧毒危险品和放射性物品管理，确保公共场所和重要设施安全。

　　五、加强社会各项事业建设，促进经济社会协调发展

　　加强社会主义新村镇建设。进一步落实农村工作“五十条”和首批九个配套文件。大力实施“科教兴农”战略，推进现代农业生态园区和海峡两岸农业合作试验区建设，发展农业龙头企业和农民专业合作经济组织，促进农业不断增效、农民持续增收。积极实施城镇化战略，完善并实施镇、村规划建设。加强中心镇（街）建设，使之成为推动农村城镇化进程的增长点、农村劳动力转移的重要载体。大力发展村民公寓社区，引导农村居民转变居住和生活方式。全面推进城乡统筹协调发展，推动交通、供水、环保等基础设施向农村延伸，教育、医疗、文化康乐等公共服务向农村覆盖。加快推进农村管理信息化建设。加强农村民主管理和民主监督，提高村委会依法管治、服务群众、化解矛盾、发展经济的能力。继续全面推广引入社会中介组织规范农村财务管理，完善农村股份合作制。引导和支持农村发展各种新型社会化服务组织，构建“共建、共享、共赢”的农村发展利益共同体。

　　加强人才队伍建设。深入实施人才强市战略，加强党政人才、企业经营管理人才、专业技术人才、技能型人才和农村实用型人才等人才队伍建设。加快培育社会工作者人才。实施“优秀人才培养引进工程”，引进紧缺人才和高层次人才，支持海外优秀人才到我市创业。加强企业博士后工作站、留学人员创业园等人才载体建设，培育壮大创新型人才队伍。进一步完善人才市场体系，创新人才激励和评价机制，营造有利于各类人才脱颖而出和干事创业的良好环境。

　　加强公共财政建设。继续推进协税护税网络建设，强化税收征管，加强和规范非税收入管理。完善公共财政体制，调整优化财政支出结构，保证科技、农业、教育、环保、社会保障、就业再就业、公共安全和城市重点基础设施等方面的资金需求，提高公共产品和公共服务供给能力。深化部门预算改革，强化国库集中支付和“收支两条线”管理，完善财政支出绩效评价制度，提高财政资金使用效益。加强政府债务监控，进一步防范化解金融、财政风险。构建政银企合作平台，加强产业政策与信贷融资政策的联动与协调，促进金融业提高服务质量和信贷融资效益；推进“信用佛山”建设，进一步改善金融生态环境。健全和完善中小企业信用担保体系，增强金融对经济增长的助推作用。

　　加快体育和其他社会事业发展。进一步完善公共体育基础设施建设，增设开放式、免费的文体活动场所。广泛开展群众性体育运动，着力提高体育竞技水平，推动体育产业加快发展。继续做好妇女儿童、人口与计划生育、统计、外事侨务、对台港澳事务、民族宗教、爱国卫生、档案、方志、气象、人防等工作，促进社会各项事业健康发展。

　　六、加强政府自身建设，建设廉政勤政善政高效政府

推进民主法制建设。认真贯彻落实市委的各项决策部署。坚持向人大及其常委会报告工作制度，自觉接受人大及其常委会监督。充分尊重和支持人民政协、各民主党派、工商联、无党派人士在政治协商、民主监督和参政议政中的作用。认真办理人大代表议案、建议和政协提案。发挥群众监督和舆论监督的作用。全面推进依法治市，抓好“五五”普法工作，提升市民法律意识。深入贯彻《行政许可法》，建立政府法律顾问制度，提高依法行政能力和水平。加强基层民主政治建设，完善政务、村（厂）务公开制度，不断拓展市民参与政务活动的渠道，推进决策的科学化、民主化和制度化。

　　加强政府廉政建设。建立健全教育、制度、监督并重的惩治和预防腐败体系，从源头上预防和治理腐败。加强反腐倡廉教育和廉政文化建设，严格执行领导干部廉洁自律各项规定，确保领导干部为民、务实、清廉。加强对权力运行的制约和监督，进一步规范土地出（转）让、政府采购、招投标等管理。继续抓好治理商业贿赂专项工作，坚决纠正损害群众利益的不正之风。加大查处案件力度，严肃惩治各类腐败分子，纯洁公务员队伍。

　　树立勤政为民作风。深入推进政府职能转变，强化社会管理和公共服务职能。确立“管理就是服务”的思想，在全市范围内创造公开、公正、公平、文明的依法办事的政务环境。坚持全心全意为人民服务宗旨，做到忠于职守、认真履职、勤政为民，真正把广大人民群众的根本利益实现好、维护好、发展好。

提高政府善政能力。加强领导干部执政能力和执政道德建设，着重提高促进科学发展、依法行政、驾驭复杂局面和协调利益关系的能力。树立正确的政绩观，切实做到情为民所系，权为民所用，利为民所谋。贯彻落实《公务员法》，抓好公务员在职培训、交流和下基层锻炼工作，完善科学的激励和淘汰机制，建设一支政治坚定、素质优良、作风过硬、充满活力的高素质公务员队伍。

　　提高政府行政效率。继续深化行政审批制度改革，进一步清理和规范行政审批事项，提高行政审批效率。加快推进全市行政审批电子网络一体化建设，开通“12345”行政服务热线，提高服务效率。实行影响行政效能行为过错责任追究，完善行政审批电子监察系统，全面推行效能监察，提升政府行政效能。

　　各位代表！做好今年政府工作，实现预期奋斗目标，使命崇高而神圣，任务艰巨而光荣。让我们高举邓小平理论和“三个代表”重要思想伟大旗帜，以科学发展观为指导，与时俱进，开拓创新，励精图治，团结奋斗，为建设产业强市、文化名城、现代化大城市与富裕和谐佛山，为创造更加美好的明天而努力奋斗！
